# Supplementary material for: Neuronal upregulation of Prospero protein is driven by alternative mRNA polyadenylation and Syncrip-mediated mRNA stabilisation
Source: Biol Open. 2020 May 4;9(5):bio049684. doi: 10.1242/bio.049684 (PMC7225087; doi:10.1242/bio.049684)
Supplement: Supplementary information [file biolopen-9-049684-s1.pdf]

SUPPLEMENTARY INFORMATION

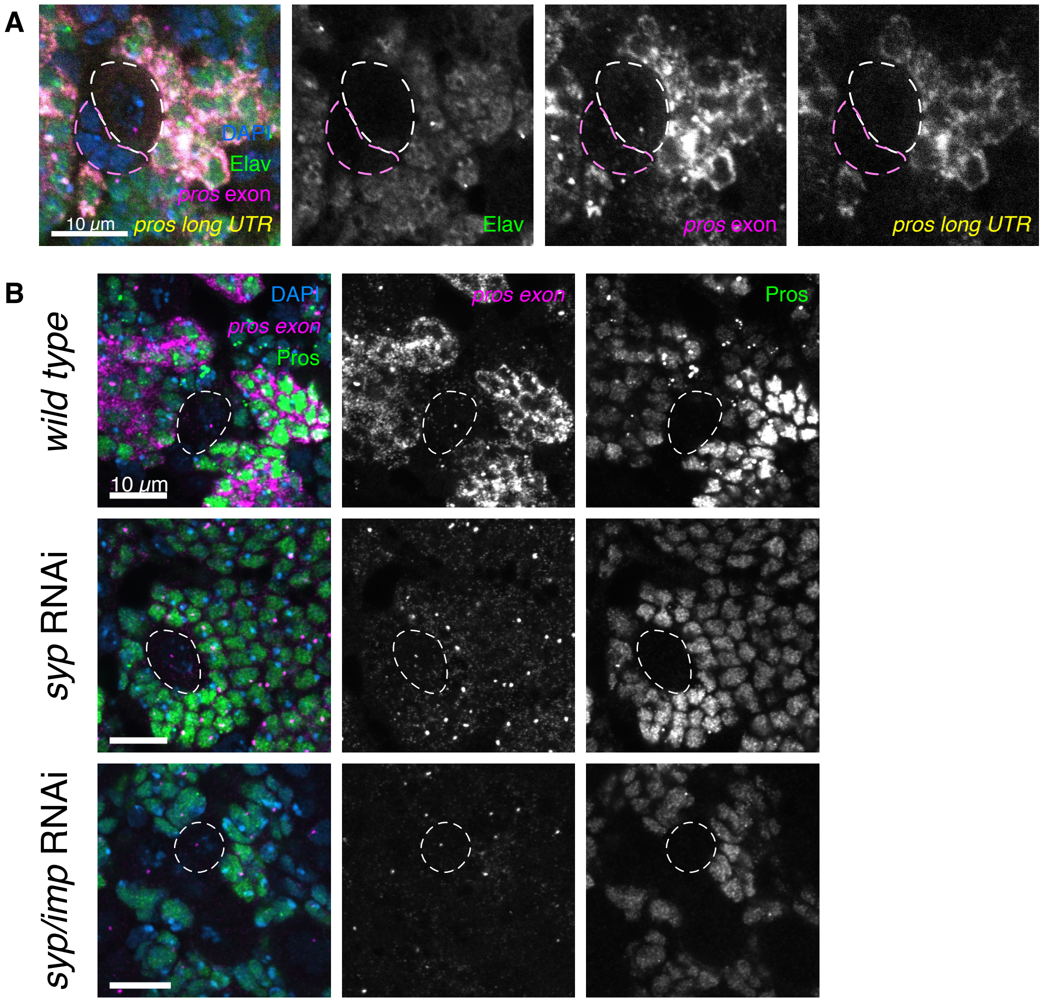

Figure S1: Syp stabilises *pros* directly, not via Imp

A Staining with Elav IF (marking post-mitotic neurons), and *pros* exon and *pros long UTR* smFISH. *pros*^{long} is only expressed in Elav+ cells, and is not expressed in the NBs (white outline) or GMCs (pink outline) **B** Brains stained with Pros protein and *pros* exon smFISH. *pros* is lost in the *syp* RNAi knockdown, and in the *syp/imp* double knockdown brains. This shows that *pros* is regulated directly by Syp, not via its downregulation of Imp. RNAi constructs are driven by insc-GAL4 (Betschinger *et al.,* 2006).

Biology Open • Supplementary information

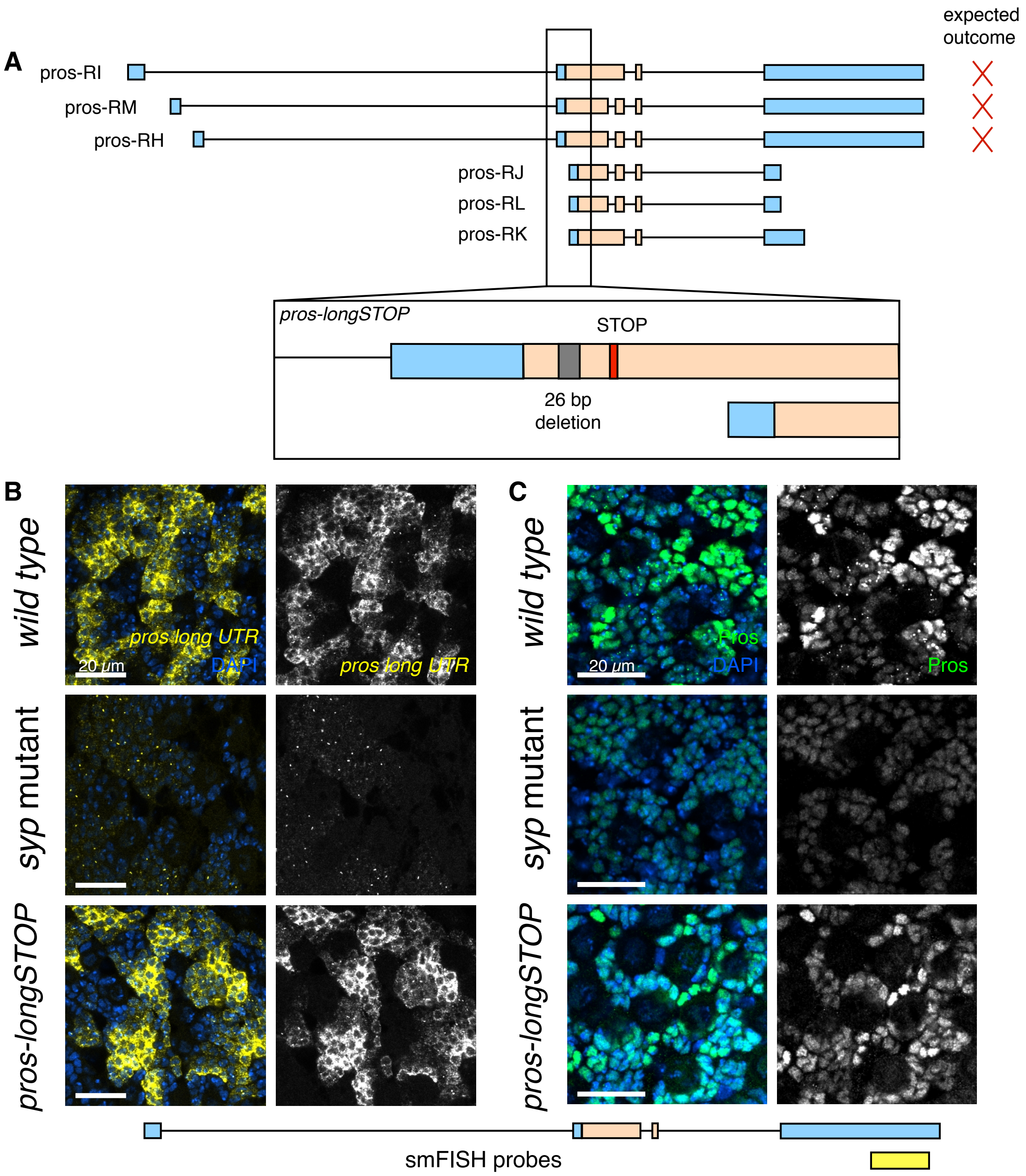

Figure S2: Introducing an NMD mutation does not remove *pros^long*

A We used CRISPR/Cas9 to make a double stranded DNA break in the upstream coding region unique to the *pros^long* isoforms. Repair by non-homologous end joining resulted in a 26 bp deletion, leading to a frame shift which introduces a premature stop codon 80 bp downstream (*pros-longSTOP*). This should produce a truncated polypeptide and induce NMD of the *pros^long* mRNA isoforms. The *pros-longSTOP* mutant was stained with **B** *pros long UTR* smFISH and **C** Pros protein. Neither *pros^long* or Pros protein were decreased compared to *wild type*.

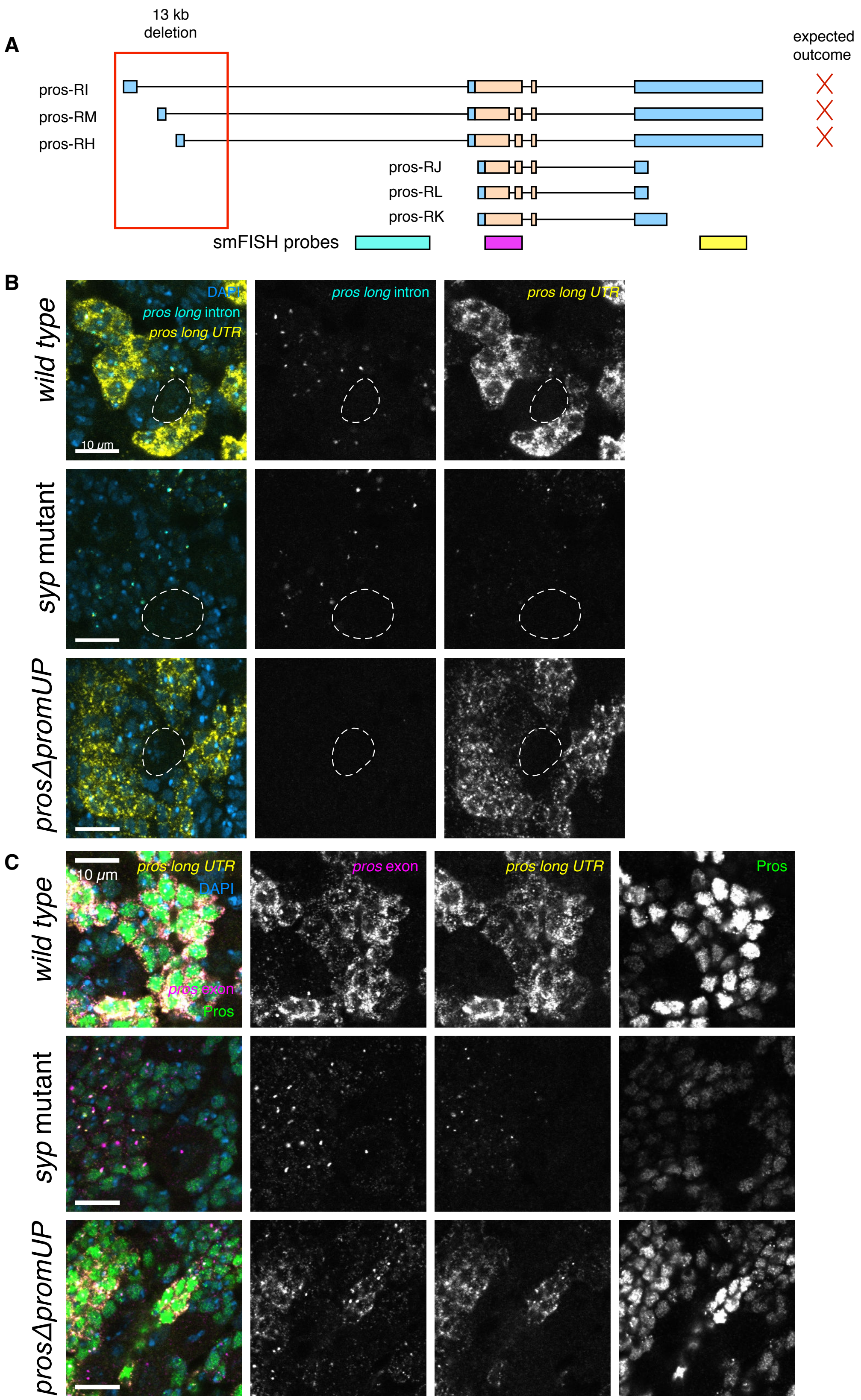

Figure S3: The deletion of the upstream *pros* promoters uncovers a new isoform
A We deleted the three upstream transcription start sites, which are annotated to produce *pros^{long}*. (*prosΔprosUP*). **B** Staining the with *pros-long* intron and *pros long* UTR smFISH showed that transcription from the upstream promoters was abolished, but low levels of *pros long* UTR signal remained. **C** Staining with Pros protein additionally showed that the neuronal upregulation of Pros protein was not disrupted.

Biology Open • Supplementary information

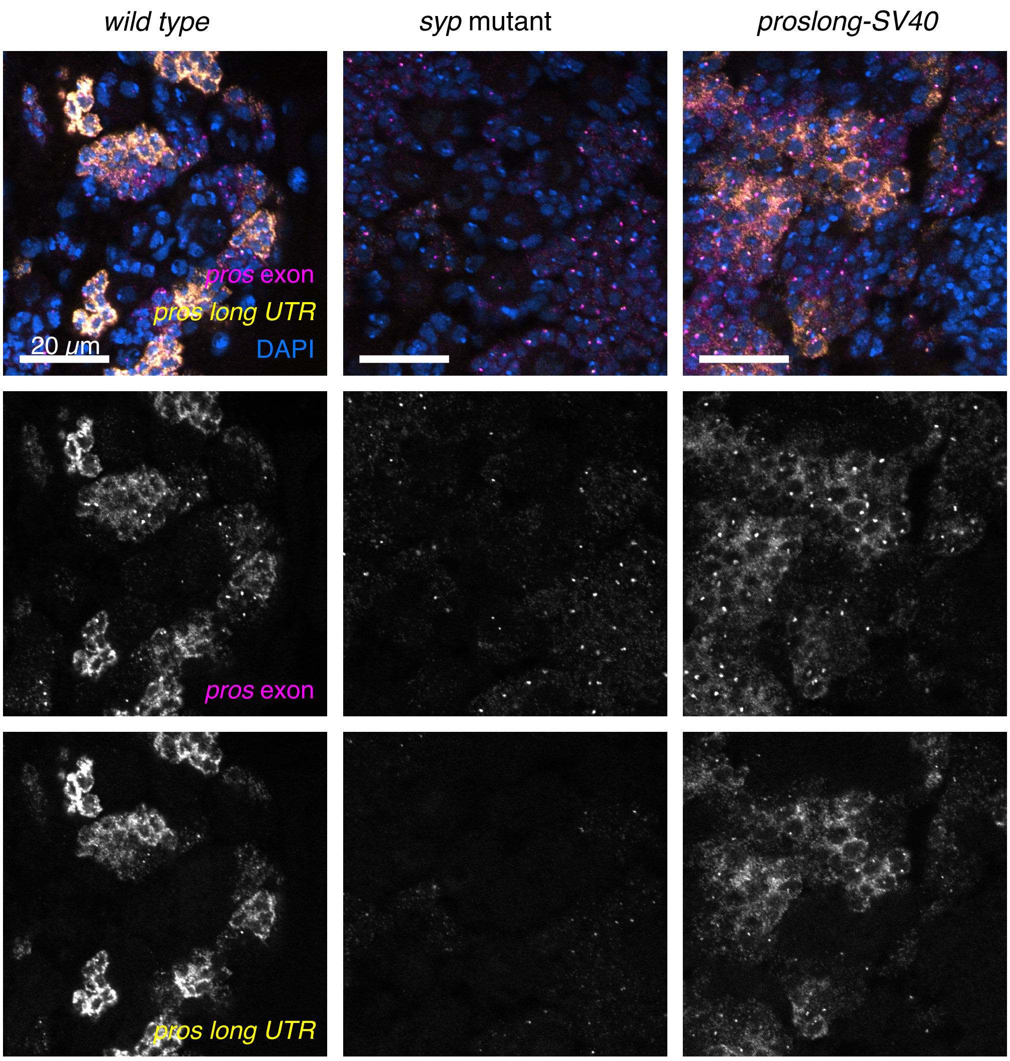

Figure S4: Insertion of an SV40 transcriptional terminator reduces, but does not abolish, *pros^{long}* expression

Staining for *pros* exon and *pros long UTR* smFISH shows a reduction in *pros^{long}* expression in the *proslong-SV40* line compared to *wild type*. However the *pros^{long}* level is not reduced as much as in the *syp* mutant.

Table S1 - Differential expression analysis of *wild type* vs *syp* mutant third instar larval brains

Click here to Download Table S1

Biology Open • Supplementary information

Table S2: Primers for generating Northern blot probes

The above primers are used to generate radioactive Northern blot probes. ORF - Open Reading Frame of *pros* transcript; UTR Long are used to generate probes that are specific to the 15 kb *pros* 3' UTR (probe LONG); UTR Short are used to generate probe that is against region that is common to all *pros* 3' UTRs (probe ALL).

Name	Sequence (5' - 3')
ORF F1	AGAAGCGCAAGCTCTACCAG
ORF R1	GTCTTGGGTTTTAGGGGCGA
ORF F2	AAGAAACCCGGCATGGACTT
ORF R2	CGTCACCATCTCCGGTCAAA
UTR Long1 F	GACGATGGTGAACGCGAAAG
UTR Long1 R	TGTGGCTGTGTTCTTGTGGT
UTR Long2 F	ATTTCCCAATCGGCGTCCTT
UTR Long2 R	TTGCCTGTCGATTGCTCTGT
UTR Short1 F	TTGGATGGGAACACCGCTAC
UTR Short1 R	GTGCTCCAAAATCGGGCTTG
UTR Short2 F	CGCAGGCCAAAGCTAAAAGG
UTR Short2 R	ACCAACGGCGAGTACAGTTT
actin F	GGTCGCGATTTAACCGACTACCTGAT
actin R	AGCAGATGTGGATCTCGAAGCAAGAG

Table S3: Primers used for RT-qPCR for Syncrip and IgG immuno-precipitation experiments

pros and housekeeping gene *rp49* was used to assess the efficiency and specificity of Syncrip binding to *pros*.

Name	Sequence (5' - 3')
rp49 F	GCTAAGCTGTCGCACAAA
rp49 R	TCCGGTGGGCAGCATGTG
pros F	TATGCACGACAAGCTGTCACC
pros R	CGACCACGAAGCGGAAATTC

Biology Open • Supplementary information

Table S4 - Sequence used to generate Stellaris® DNA probe sets

Click here to Download Table S4

Table S5: gRNA constructs

Guide RNA constructs used to produce the *pros*^{long} deletion lines.

***pros* deletion lines**	**gRNA**	**Use**
pros-longSTOP	GTGTGACCGTTGCTGCTCGG_CGG	NMD mutant
prosΔpromUP (upstream)	TTCCTACTAACTCATGCACA_TGG	Upstream promoter deletion, upstream cut
prosΔpromUP (downstream)	GTTGGGCTACTAGAACTACA_AGG	Upstream promoter deletion, downstream cut
prosΔUTR (upstream)	TTTAGTGAGATGTGTGAAGG_TGG	Long UTR deletion, upstream cut. SV40 insertion site.
prosΔUTR (downstream)	ACTAAAAATGTGTAATGGAA_TGG	Long UTR deletion, downstream cut

Biology Open • Supplementary information

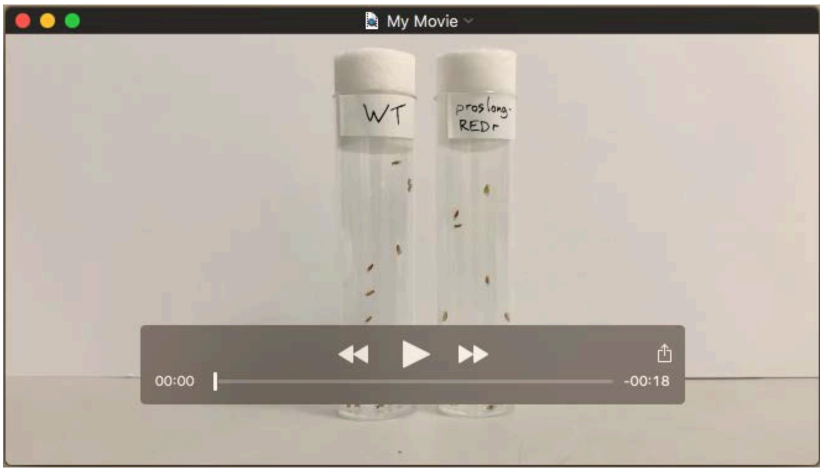

Movie 1 - *proslong-REDr* flies exhibit defects in their activity

1-3 day old mixed sex flies were transferred to empty vials and allowed to acclimatise for two hours without disturbance. The vial on the left contains *wild type* flies and the vial on the right contains *proslong-REDr* homozygous flies. After disturbance, by tapping the vial to bring all flies to the bottom, the *wild type* flies immediately crawl up the sides of the vial. However the activity of the *proslong-REDr* flies is impaired and the flies remain at the bottom of the vial.

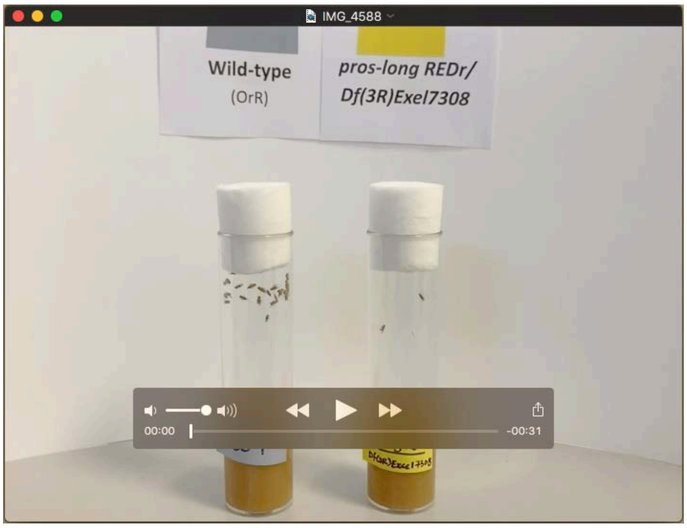

Movie 2 - hemizygous *proslong-REDr* flies phenocopy the homozygous *proslong-REDr* adult activity defect

1-3 day old mixed sex flies were transferred to empty vials and allowed to acclimatise for two hours without disturbance. The vial on the left contains *wild type* flies and the vial on the right contains *proslong-REDr/Df(3R)Exel7308* flies. The deficiency (Df(3R)Exel7308) includes the entire *pros* gene region. After disturbance, by tapping the vial to bring all flies to the bottom, the *wild type* flies immediately crawl up the sides of the vial. The activity of the hemizygous *proslong-REDr* flies is impaired, similarly to the *proslong-REDr* flies in Movie S1. This result suggests that the phenotype of *proslong-REDr* is not due to a CRISPR off-target effect.
